# Calciphylaxis: Successful Management of a Rare Complication of Chronic Kidney Disease in Two Patients

**DOI:** 10.1155/2019/1630613

**Published:** 2019-06-17

**Authors:** Ibtissam Fares, Tarik Bouattar, Hadja Moussokoro Kone, Hajar Benzouina, Meryem Benbella, Naima Ouzeddoun, Rabia Bayahia, Loubna Benamar

**Affiliations:** ^1^Nephrology Dialysis and Transplantation Department, Ibn Sina Teaching Hospital, Rabat, Morocco; ^2^Faculty of Medicine and Pharmacy of Rabat, Morocco

## Abstract

Calciphylaxis, or calcification uremic arteriolopathy, is a rare disease thought to occur due to arteriolar calcifications of the dermis and is responsible for ischemia with cutaneous necrosis and painful panniculitis. Its mechanism remains poorly understood which makes its management challenging and difficult to standardize. We report our management of two patients diagnosed with calciphylaxis. In one patient, calciphylaxis was mentioned upon admission given the context of preexisting secondary hyperparathyroidism and the existence of multiple risk factors. In both patients, the diagnosis was confirmed histologically. Our two observations highlight the difficulty of the diagnosis and the complexity of the therapeutic management that has been personalized according to patient characteristics and clinical evolution. Several therapeutic means can be implemented once the diagnosis is made; nevertheless, its prognosis remains pejorative despite the therapeutic advances. Broad debridement, good phosphocalcic balance control, and the correction of the risk factors top the list of any therapeutic strategy. One of the major challenges of the therapy is normalizing the calcium-phosphate balance. Thus, Cinacalcet and sodium thiosulfate appear to be promising treatments.

## 1. Background

Calciphylaxis, or calcific uremic arteriolopathy, is a rare disease with a poor prognosis. The mechanism involves arteriolar calcifications of the dermis, responsible for ischemia, with cutaneous necrosis and painful panniculitis [1].

Risk factors are multiple: female sex, dialysis, advanced age, diabetes, obesity, undernutrition, antivitamin K, and poor phosphocalcic balance control [2, 3].

The treatment is primarily based on managing the wounds, eliminating all the possible precipitating factors of ectopic calcification, administering agents capable of inhibiting the process of calcification, and controlling risk factors to avoid recurrences.

This disease is associated with high mortality ranging between 30% and 80% depending on the comorbidities and the configuration of cutaneous involvement. In recent years, mortality seems to have improved; especially, since the pathology is better known, the action is taken more quickly and some treatment alternatives are available [4].

We report a successful evolution of two patients with calciphylaxis managed in our department.

## 2. Cases Presentation

### 2.1. Patient 1

**Figure 1:**
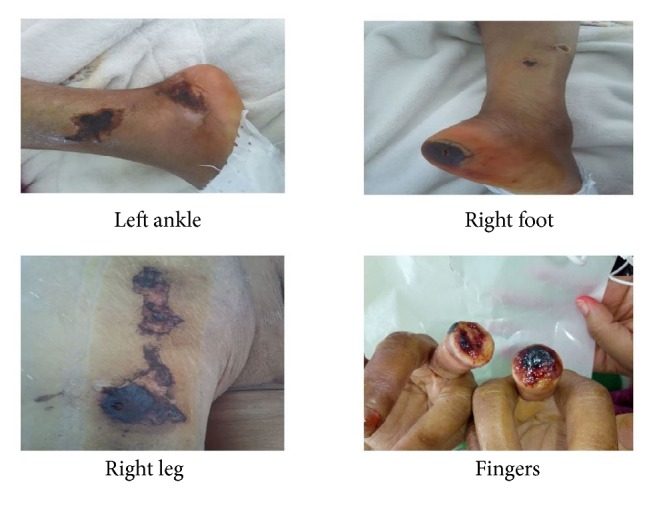
Topography and aspect of Calciphylaxis lesions of patient 1.

A 56-year-old woman with a 27-year history of type 2 diabetes mellitus being poorly controlled the last 3 years and high blood pressure under angiotensin-converting enzyme inhibitor (ACEI) for 4 years was diagnosed with end-stage kidney disease presumably due to diabetic nephropathy. After three years of thrice-weekly hemodialysis treatment (with a single-pool Kt / V at 1.27 and dialysate Ca at 1.5 mmol/L), our patient presented with necrotic and painful extremities skin lesions (Figure 1). The clinical examination found a patient in good general health with present and symmetrical peripheral pulses. Its biological assessment revealed phosphocalcic balance disorders with an elevated parathormone (PTH) and alkaline phosphatase (PAL) at 919 pg /ml and 348 UI /l, respectively, a calcium level at 2.2 mmol / l under calcium carbonate, a normal serum phosphorus at 1.03 mmol / l, a vitamin D deficiency at 14.2 ng / ml, and normocytic normochromic anemia. Dosage of prothrombotic factors (C and S proteins, antiphospholipid antibodies, anticardiolipin antibody, anti-b2 glycoprotein 1 antibody, circulating anticoagulant, and cryoglobulinemia) was normal. Cervical ultrasound has found bilateral parathyroid nodules. X-rays of the skeleton showed bone demineralization with extensive calcification of the vessels. The patient initially received symptomatic treatment with an opioid analgesic (Tramadol sometimes associated with Nefopam), blood transfusion, and erythropoietin to correct anemia.

**Figure 2:**
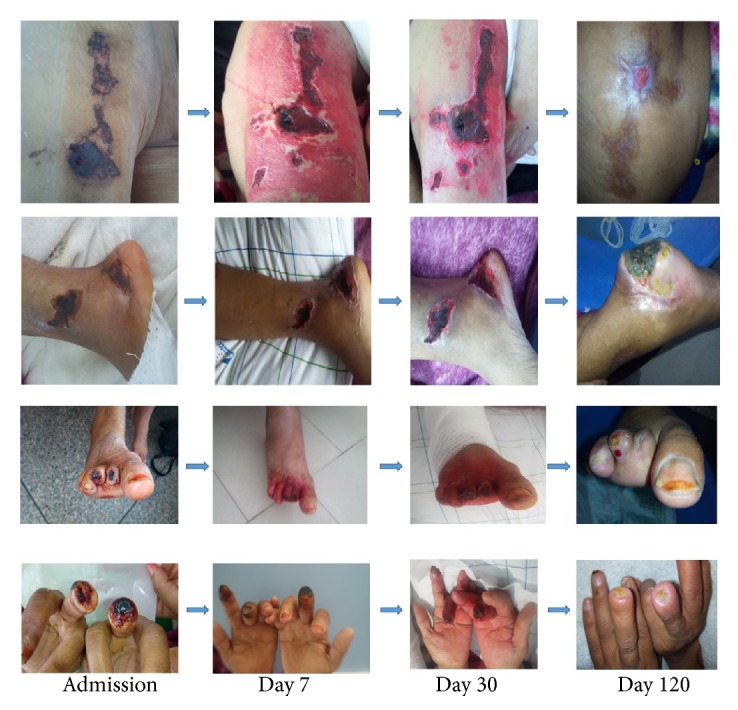
Evolution of Calciphylaxis lesions over 4 months of treatment in patient 1. Necrosectomy could not be performed at the heels because of the risk of injury to the Achilles tendon.

She underwent a wide debridement of the necrotic cutaneous lesions whose anatomopathological examination returned in favor of a calciphylaxis. Once the diagnosis was established, the patient first benefited from a parathyroidectomy in order to correct the phosphocalcic balance. One week after parathyroidectomy, the patient had asymptomatic hypocalcemia at 1.74 mmol/l, following which she was dialyzed with a dialysate rich in calcium 1.75 mmol/l and given calcium supplementation based on calcium carbonate. 3 weeks later, its balance sheet improved significantly with a PTH at 432 pg/ml, a serum calcium level at 2.29 mmol/l, and a hypophosphatemia at 0.64 mmol / l. In addition, the patient received several hyperbaric oxygen therapy sessions that started from the second week of her admission and had been maintained until the fourth month with a total of 36 sessions. Sodium thiosulfate perfusions (25g three times a week at the end of each hemodialysis session) were also administered to our patient that started from the fourth week to the sixteenth week with a total of 36 bottles. No adverse effects have been reported. Local care was performed daily until complete healing of the lesions (Figure 2).

### 2.2. Patient 2

**Figure 4:**
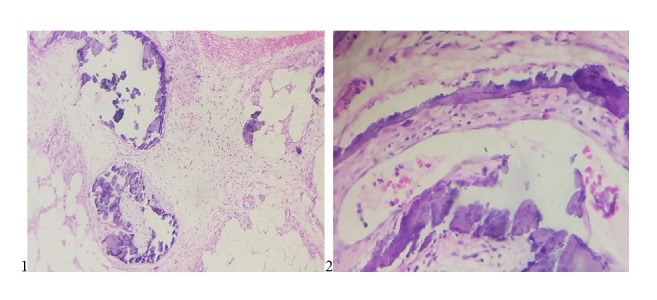
(1) A skin biopsy and adipose tissue in patient 2 with foci of necrosis, calcification of arteriolar walls, and necrotizing panniculitis in the context of a uremic calcifying arteriopathy. (2) Intimal thickening and fibrin thrombi in patient 2 skin biopsy.

**Figure 5:**
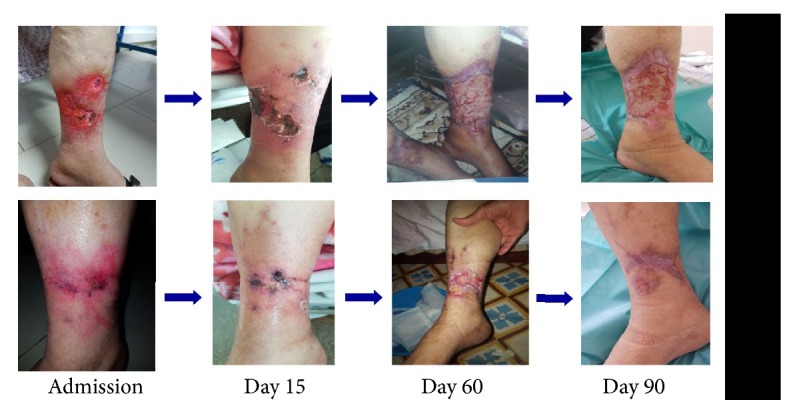
Evolution of Calciphylaxis lesions over 3 months of treatment in patient 2.

A 69-year-old woman with a long history of arterial hypertension under ACEI, complicated by end-stage kidney disease was placed on automated peritoneal dialysis (APD) for 21 months with a KT/V urea at 1,69. The patient was also under calcium carbonate for secondary hyperparathyroidism diagnosed during her follow-up (a PTH at 780 pg/ml). The patient was consulted for erythematous, necrotic, and painful skin lesions of her right leg (Figure 3.1). The clinical examination found inflammatory signs with redness and pain around these lesions. Peripheral pulses were present and symmetrical. Her body mass index was at 28,3 kg/m^2^. The lesions worsened and spread to the contralateral leg within 5 days (Figure 3.2). CT angiography did not indicate stenosis of the vascular axes but showed diffuse calcifications that extended to the lower limbs. Calciphylaxis was mentioned in view of the different risk factors present in our patient, as well as the quite telling clinical presentation occurring in a known context of secondary hyperparathyroidism. The initial treatment consisted of the correction of anemia with erythropoietin to optimize the tissue perfusion as well as analgesics to manage the pain. Parathyroidectomy was performed as soon to control the disturbances of the phosphocalcic balance and the PTH levels decreased to 417 pg/ml the 2^nd^ day after parathyroidectomy. A concomitant biopsy of skin lesions confirmed the already mentioned diagnosis of calciphylaxis (Figure 4). Necrosectomy with local care and optimization of dialysis parameters were also implemented. A treatment based on sodium thiosulfate and hyperbaric oxygen was proposed to our patient but was not performed due to a lack of her financial resources. Fortunately, the complete healing of the lesions was obtained by 4 months (Figure 5).

## 3. Discussion

Calciphylaxis was described in 1968 by Anderson et al. in a context of secondary hyperparathyroidism with hypercalcemia [9]. Since then, it has been more frequently observed in patients with chronic kidney disease with a frequency ranging from 1 to 4% according to the studies [1, 10].

The average age of onset is between 50 and 60 years with a clear female predominance. The risk factors for calciphylaxis commonly cited in the literature are as follows: the number of years of dialysis, the presence of phosphocalcic balance abnormalities (including hypercalcemia, hyperphosphatemia, and secondary hyperparathyroidism, favoured by calcium supplementation and vitamin D deficiency), the use of antivitamin K or corticosteroids, the presence of diabetes, obesity, female sex, hypoalbuminemia, and liver diseases. Thrombophilia also appears to be one of the predisposing factors for calciphylaxis [7, 9, 10].

Both of our patients had an obvious predisposing ground. Besides the female sex, dialysis, and secondary hyperparathyroidism risk factors were diabetes in the first patient and the overweight in the second.

The pathogenesis of calciphylaxis remains incompletely understood. It has been advanced that these lesions are secondary to calcification of the small vessel's wall, with fibrosis and endovascular thrombosis, leading to tissue ischemia and necrosis of the dermis and hypodermis.

The development of calciphylaxis goes through two phases: a silent phase where vascular calcifications are formed in the media with intimal hyperplasia of dermal and hypodermic arterioles, and then a phase of microcirculatory decompensation, which corresponds to thrombosis ischemia most often [8].

The diagnosis of calciphylaxis is based on a set of arguments essentially clinical [3, 8].

Clinical signs include skin lesions which start as subcutaneous indurated nodules or plaques extremely painful (a pain disproportionate to the cutaneous lesion) accompanied by livedo reticularis, often initially labeled dermis-hypodermis. The evolution is done in a few days towards the formation of superficial and then deep ulcerations leading to the constitution of a blackish eschar, always strongly painful with centrifugal extension [11].

This suggestive clinical presentation associated with risk factors should rapidly evoke the diagnosis and initiate early therapeutic management to avoid serious infectious and ischemic complications, which can lead to death [1–3, 8, 10]

Indeed, in patients presenting with plaques only, the mortality rate was 33% at six months. Once ulceration develops, the mortality rate increased to above 80% [12].

Two strong arguments in favor of a diagnosis of calciphylaxis are the presence of pulse distal and lack of neuropathy [13].

Imaging tests may contribute to the diagnosis of calciphylaxis. A simple X-ray of the affected area can demonstrate the presence of calcifications of the vessels of medium-small caliber.

The biological context is very particular. In the vast majority of cases, there are secondary hyperparathyroidism and profound disorders of phosphocalcic metabolism with hypercalcemia and hyperphosphatemia. The thrombophilia assessment is useful and must be performed according to antecedents [8, 11].

Although in the context of the clinical signs biological and radiological features guide the diagnosis, the biopsy of skin lesions would confirm the diagnosis of calciphylaxis [4, 8].

Pathologically, this entity is characterized by the existence of calcifications of the media of deep and hypodermic dermal arterioles, hypertrophy of the intima, vascular thrombi formation, adipose tissue calcification, and infiltration by inflammatory cells [1, 8, 10].

Given the low level of evidence and the lack of randomized studies in this field, optimal treatment is far from being standardized. It involves a medical and surgical approach.

Management will, therefore, aim at the restoration of phosphocalcic balance, effective detoxification of affected areas, and the elimination of various risk factors [3, 8, 10].

Surgical wound cleaning is the first step in the management of this disease. McCarthy et al. reported in their retrospective monocentric study that a lack of surgical debridement was associated with a lower 6-month survival and significantly poorer survival for the entire duration of follow-up [6].

Other measures would be essential, notably the management of ulcerations (debridement, directed cicatrization, and broad-spectrum antibiotherapy depending on the evolution) and the optimization of healing (nutritional management, pressure ulcer prevention, and physiotherapy) [3].

Some authors advocate hyperbaric oxygen therapy in addition to previous treatments to accelerate wound healing [5].

The effective treatment of pain with analgesics using opioids is often appropriate. In our cases, opioids were used essentially during local care and dressing changes where the pain was at its peak.

The correction of anemia (by erythropoietin or transfusion of red blood cells) and the optimization of dialysis parameters (daily dialysis for patients on hemodialysis) allow better tissue oxygenation, essential for good healing of lesions [3].

It is accepted by several authors that the normalization of calcium phosphate balance is the primary variable for the treatment of calciphylaxis lesions [6].

Optimal dialysate poor in calcium and stopping the intake of calcium and alpha hydroxylated derivatives of vitamin D help to fight against the phenomena of calcification [3].

The control of hypoprothrombinemia by calcimimetics is strongly recommended or, more radically, parathyroidectomy in case of secondary hyperparathyroidism. McCarthy et al. found that, in patients with stage 5 / 5D chronic kidney disease (CKD), subtotal parathyroidectomy (performed only in patients with secondary hyperparathyroidism) was associated with better 6-month survival and overall survival [6].

Cinacalcet represents an interesting alternative for controlling hyperparathyroidism and decreasing the phosphocalcic product especially for patients with surgical contraindications [14].

In our patients, we used parathyroidectomy for the rapid treatment of secondary hyperparathyroidism and better control of the calcium phosphate balance. However, we have used a calcium carbonate substitution given the hypocalcemia threatening after parathyroidectomy. In addition, we did not correct the hypovitaminosis D during the 4 months of evolution.

Sodium thiosulfate, a calcium ion chelator, is a specific treatment that proved its worth in slowing calcification phenomena. Its mechanism of action is based on its ability to form soluble complexes with many metals and minerals, as well as being a possible effect as a vasodilator, antioxidant, and direct inhibitor of the calcification process [1–3, 6–8].

Bisphosphonates are the drugs of choice in the management of bone disorders including osteoporosis, Paget's disease, multiple myeloma, bone cancer, and cancer-induced hypocalcemia. It has shown response mainly in dystrophic calcifications of dermatomyositis and systemic sclerosis by reducing calcium turnover and resorption [15]. Furthermore, because of an additional property of inhibiting calcium phosphate crystal formation, bisphosphonates were used as inhibitors of ectopic calcification in two different types of vascular disease, atherosclerosis, and calciphylaxis [16, 17]. We do not have the experience of the use of bisphosphonates in our cases.

The calciphylaxis is one of the most feared complications of patients with CKD and dialysis. Its prognosis is very poor with mortality that reached 100% at 12 months as reported by Jean et al. [3]

Despite being improved in recent years by a better understanding of the pathology, the mortality remains high ranging between 33% and 80% mainly related to sepsis in most than half of the cases [8, 18].

The severity of calciphylaxis is related to the underlying condition of the patient (obesity, diabetes, and arteritis), diagnosis delay, the extent of the lesions, and the topography of the affected area with a better prognosis for distal forms [1, 3, 8, 18].

Our little experience in the management of calciphylaxis made us more optimistic toward this feared disease. We believe that the creation of a national registry for calciphylaxis and referral of patients to centers with experience in the management of calciphylaxis may help to carry out studies on a larger number of patients and then may help in improving the knowledge of calciphylaxis and therefore the survival of patients facing this disease.

Table 1 includes the nonexhaustive list of different therapies suggested to improve calciphylaxis in our patients and their mechanism of action.

## Figures and Tables

**Figure 3:**
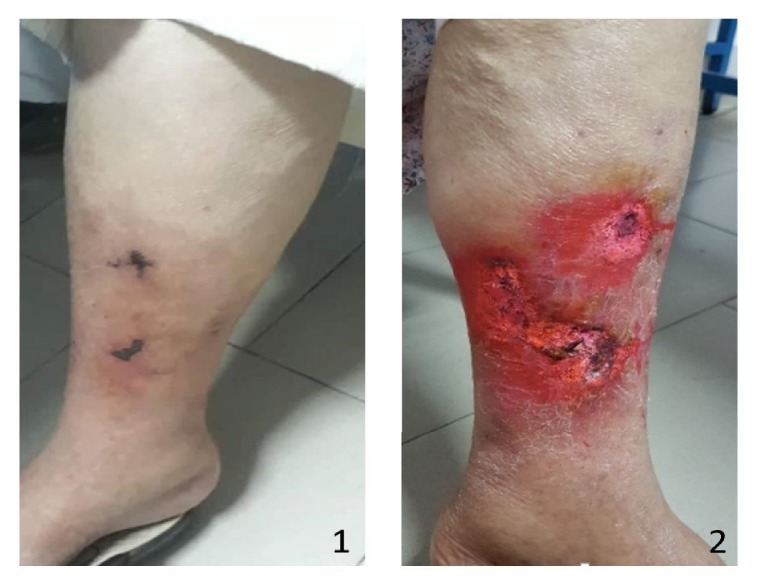
Calciphylaxis lesions at the admission of patient 2 (A: right leg, B: left leg).

**Table 1:** Therapies that have been suggested to improve calciphylaxis and their mechanism of action.

Therapies	Mechanism of action
Hyperbaric oxygen therapy	Promote tissue oxygenation [5]
Correction of anemia	Promote tissue oxygenation [3]
Stopping alpha-hydroxylated vitamin D derivatives	Slow down calcification phenomena [1, 3]
Dialysate calcium 1.5 mmol / L	Slow down calcification phenomena [3]
Parathyroidectomy	Slow down calcification phenomena [1, 3, 6]
Sodium thiosulfate	Slow down calcification phenomena [1–3, 6–8]
Debridement	Ulcerations treatment [1, 6]
